# Supplementary material for: Ac/Ds-like Transposon Elements Inserted in ZmABCG2a Cause Male Sterility in Maize
Source: Int J Mol Sci. 2025 Jan 15;26(2):701. doi: 10.3390/ijms26020701 (PMC11766044; doi:10.3390/ijms26020701)
Supplement: Supplementary file 1 [file ijms-26-00701-s001.zip › ijms-3345929-supplementary.pdf]

Supplementary

Ac/*Ds*-like Transposon Elements Inserted in *ZmABCG2a* Cause Male Sterility in Maize

Le Wang [1], Saeed Arshad [1], Taotao Li [1], Mengli Wei [1], Hong Ren [2], Wei Wang [2], Haiyan Jia [1], Zhengqiang Ma [1] and Yuanxin Yan [1,3,*]

[1] State Key Laboratory of Crop Genetics and Germplasm Enhancement, Nanjing Agricultural University, Nanjing 210095, China; 2020201074@stu.njau.edu.cn (L.W.); maliksaeedawan@hotmail.com (S.A.); 2020101012@stu.njau.edu.cn (T.L.); wmengli@foxmail.com (M.W.); hyjia@njau.edu.cn (H.J.); ww1980666@126.com (Z.M.)
[2] Guizhou Institute of Upland Food Crops, Guizhou Academy of Agricultural Sciences, Guiyang 550001, China; rhong666@163.com (H.R.); wwmaize@126.com (W.W.)
[3] Jiangsu Collaborative Innovation Center for Modern Crop Production, Nanjing Agricultural University, Nanjing 210095, China
* Correspondence: yuanxin.yan@njau.edu.cn

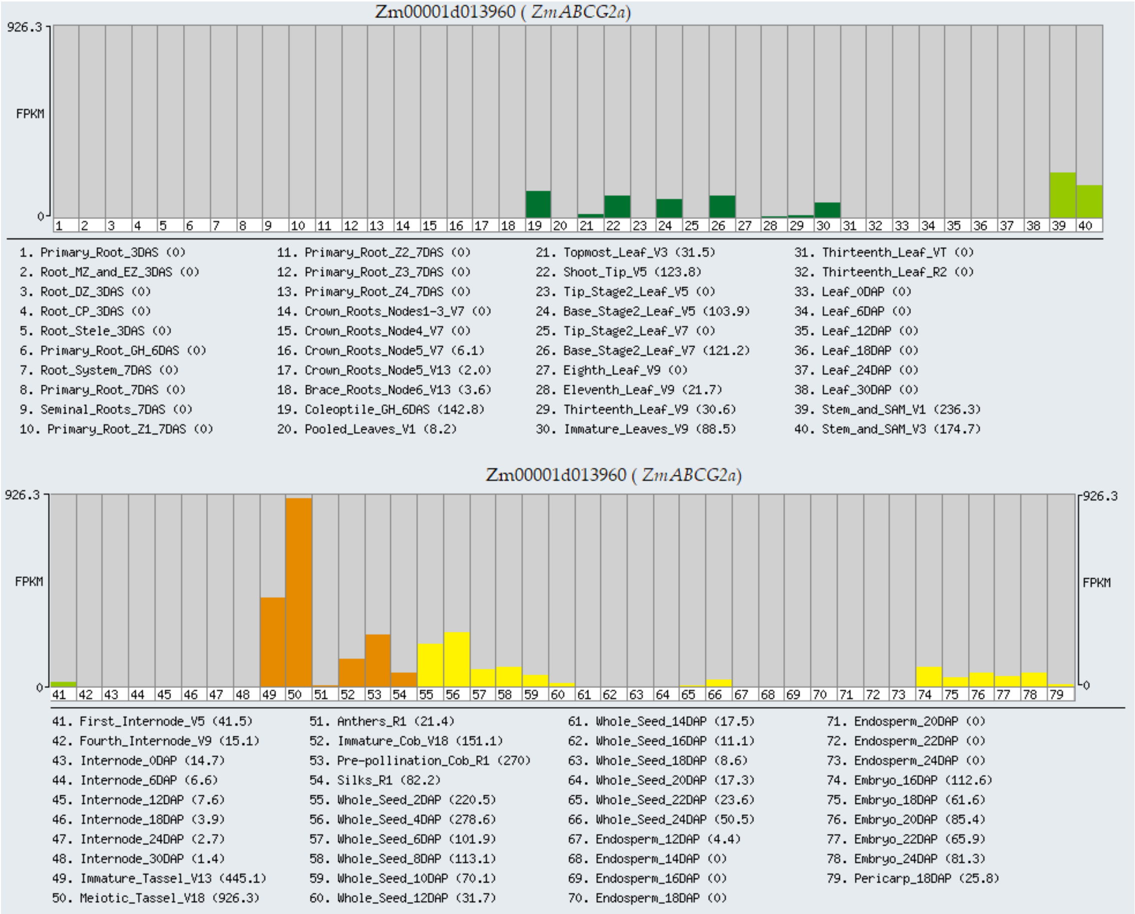

Figure S1. The tissue-specific expression pattern of Zm00001d013960 (*ZmABCG2a*) in the life cycle of maize (www.maizegdb.org).

Table S1. Sequencing reads and alignment to genome of BSR-seq samples of mutant and WT

Category	Wild Type	*ms*-N125*
reads number	239,514,266	206,742,672
reads length	150 bp	150 bp
total base	35,927,139,900	31,011,400,800
Q20 (%)	96	95
Q30 (%)	93	94
GC (%)	68	66
pair reads	119,757,133	103,371,336
aligned concordantly 0 times	25,303,409 (21.3%)	23,779,394 (23%)
aligned concordantly 1 time	77,356,464 (64.59%)	64,789,954 (62.68%)
aligned concordantly >1 times	17,097,260 (14.28%)	14,801,988 (14.32%)
total alignment ratio	86.66%	86.23%

Note: Reads number: the number of reads obtained by sequencing; Total base: the total number of bases obtained by sequencing; Q20 Content: proportion of bases with accuracy greater than 20 (99%) to the total number of bases; Q30 Content: proportion of bases with accuracy greater than 30 (99.9%) to the total number of bases; GC percentage: percentage of Guanine (G) and Cytosine (C) bases in total bases; Pair reads: reads pairs obtained by double-end sequencing;

aligned concordantly 0 times: proportion of data not compared to the reference genome; aligned concordantly 1 time: the proportion of data uniquely compared to the reference genome; aligned concordantly>1 time: ratio of data from multiple comparisons to reference genomes.

Table S2. The Primers for genetic fine-Mapping.

Name	Loci	Forward (5′→3′)	Reverse (5′→3′)	Mutant (bp)*	B73 (bp)*
2150	21501971	CGGGTGCCTCTCATAGTCAT	CCGAAGAACGAGCTGAAAAC	76	143
2311	23110153	TGAGGCTGGTAAAACGAACTG	GGTTGGAGGAGGCCATACAT	131	97
2518	25181091	GGAGTGTATGGCAGGGAAAG	ACCCACGCCTATAGTGTTGC	130	160
2566	25663549	AGCAAAAACTCCAGAGCAGAG	GCTAGCGAGCTCTAGTTCAACA	189	126
2585	25857609	GTCCACGGTCCAGCACATA	TGATCTAATGTGAATTTTGGGC	128	109
2592	25924621	GGTTGTGGATGTTTCGATCC	GCCCTGTAGAGCTCACTTGC	149	164
2622	26225988	GCGAGCAAAAAGCCTCTCTA	GAGAGAGAGAGAGAGCGCGA	158	169
2633	26337459	GTCTGGTGGATGCGGATG	AGTCAACTTTCATCTGCCCA	264	81
2643	26434596	AAAGAAACGAGAAAAATTCCGA	GAGGGGTATACCGAGGGTGA	409	87
2671	26719928	CTCCAATGTGTTCGGCCA	GACGTTCGTTTCGTCTGTTG	141	96
2778	27780855	TGACTGTGCAAGAAAGCTCTG	TGCCTGCAGCTATAATATGAAGA	154	124
2806	28060714	AACGACGCCGAGTCCTTC	TCAGCTTCAGTGTCCGAGC	163	137
3071	30715016	GTGCAAACATAGGGGGTGAT	CATCAGGATCTGGCTTGGAT	176	111
3571	35715016	CAGCACACGAGTCTAGCATCA	TCCTGTGGGAATCTCCTTTG	206	131

*bp = base pairs

Table S3. Genotypes and Phenotypes of the individuals of F_2 and F_3 Populations for *ms*-N125* Mapping.

	Markers								Phenotypes	
	2150	2311	2518	2566	2778	2806	3071	3571	Trait	Fertile (F)/Sterile (S)
F_3-79	2	0	0	0	0	0	0	0	0	S
F_3-375	2	0	0	0	0	0	0	0	0	S
F_3-434	2	0	0	0	0	0	0	0	0	S
F_3-562	2	0	0	0	0	0	0	0	0	S
F_2-562	2	0	0	0	0	0	0	0	0	S
F_2-600	2	0	0	0	0	0	0	0	0	S
F_2-631	0	1	1	1	1	1	1	1	1	F
F_2-486	0	1	1	1	1	1	1	1	1	F
F_2-597	0	1	1	1	1	1	1	1	1	F
F_3-136	0	1	1	1	1	1	1	1	1	F

F_3-307	0	1	1	1	1	1	1	1	1	F
F_3-380	0	1	1	1	1	1	1	1	1	F
F_2-616	1	1	0	0	0	0	0	0	0	S
F_2-651	1	1	0	0	0	0	0	0	0	S
F_2-622	1	1	0	0	0	0	0	0	0	S
F_2-696	1	1	0	0	0	0	0	0	0	S
F_3-323	0	0	1	1	1	1	1	1	1	F
F_2-546	0	0	0	1	1	1	1	1	1	F
F_2-130	0	0	0	1	1	1	1	1	1	F
F_2-709	1	1	1	1	0	0	0	0	1	F
F_3-366	0	0	0	0	1	1	1	1	0	S
F_2-111	1	1	1	1	1	0	0	0	1	F
F_3-124	1	1	1	1	1	0	0	0	1	F
F_3-24	0	0	0	0	0	0	1	1	0	S
F_2-467	0	0	0	0	0	0	1	1	0	S
F_2-24	1	1	1	1	1	1	1	0	1	F
F_2-31	1	1	1	1	1	1	1	0	1	F

Note: F_2 and F_3 populations were planted at the Baima Experimental Station of Nanjing Agricultural University in 2020 and 2021. 0 represents the *ms*-N125* allele, 1 represents the heterozygous genotype, 2 represents the B73 allele, and -1 represents deletion.

Table S4. Genotypes and Phenotypes of the individuals of BC_1F_2 and BC_2F_2 Populations for *ms*-N125* Mapping.

Markers									Phenotypes	
	**2566**	**2585**	**2592**	**2622**	**2633**	**2643**	**2671**	**2778**	**Trait**	**Feritle (F)/Sterile (S)**
BC_1-50	0	-1	1	1	1	1	1	1	1	F
BC_1-91	0	0	0	0	0	0	0	1	0	S
BC_2-1265	0	0	0	0	0	0	0	1	0	S
BC_2-9	0	0	0	0	0	0	0	2	0	S
BC_1-1383	0	0	0	0	0	0	1	1	0	S

BC_1-89	0	0	0	0	0	0	1	1	0	S
BC_1-1132	0	0	0	0	0	0	2	2	0	S
BC_1-1171	0	0	0	0	0	0	2	2	0	S
BC_2-189	0	0	0	0	1	1	1	1	1	F
BC_2-57	0	0	0	0	1	1	1	1	0	S
BC_1-161	0	0	0	0	2	2	2	2	0	S
BC_1-10	0	0	0	1	1	1	1	1	1	F
BC_2-117	0	0	0	2	2	2	2	2	2	F
BC_1-150	0	0	0	2	2	2	2	2	2	F
BC_2-100	1	0	0	0	0	0	0	0	0	S
BC_1-8	1	1	0	0	0	0	0	0	0	S
BC_2-114	1	1	1	1	0	0	0	0	0	S
BC_2-231	1	1	1	1	0	0	0	0	1	F
BC_2-97	1	1	1	1	1	1	1	0	1	F
BC_2-1226	1	1	1	1	1	1	1	0	1	F
BC_2-1155	2	0	0	0	0	0	0	0	0	S
BC_2-7	2	2	2	2	2	0	0	0	0	S
BC_2-31	2	2	2	2	2	0	0	0	0	S
BC_2-24	2	2	2	2	2	0	0	0	0	S
BC_2-191	2	2	2	2	2	2	2	0	2	F

Note: BC_1F_2 and BC_2F_2 populations were planted at the Baima Experimental Station of Nanjing Agricultural University in 2021 and 2022. 0 represents the *ms*-N125* allele, 1 represents the heterozygous genotype, 2 represents the B73 allele, and -1 represents deletion.

Table S5. Sequencing Primers of Candidate Gene.

Candidate Gene	Primer Name	Forward (5′→3′)	Reverse (5′→3′)	Length (bp)*
Zm958	Zm958-1	AGTCAGACAAGGCAAAGCGA	AGCTATCGCAAAGACAATGCC	1839
	Zm958-2	TTTCTACGCGCTTTGCTGTG	TAGGGGTGGTAATGGATCGC	1439
	Zm958-3	AGGAGGAGTGTTGCTCATGC	CTACGGTTTTCTGGCTCCCA	1640

Zm959	Zm959-1	CCCCATAACGTGTGCCTCAT	TGCATATGACAATCTAGAGCCG	1360
	Zm960-1	GTGAAGCTTAGCCATTCTTGCG	GCACATTGTTTTGCATCATCGG	1214
Zm960	Zm960-2	AGTGGCCTTGACAGGTACTTT	TATAGTCCCGATGCAAAGCGT	916
	Zm960-3	CTCGCAGAAAGGAACGGTGC	CTATTCCAATCCGGAACCAATCC	1497

*bp = base pairs

Table S6. Genotyping Primers for *zmabcg2a Mutant.**

Gene	Forward (5′→3′)	Reverse (5′→3′)	Length (bp)*
***Zm960* (*zmabcg2a**)**	CATCGTGGAGATGGGGCTG	AATGGAGCAGCTGGCGCTTA	865

*bp = base pairs

Table S7. Lipid Counts.

Lipid Class	Full Name	Lipid Species	Lipid Count
AEA	N-Acylethanolamine	AEA(18:2);AEA(16:0);AEA(13:0)	3
AcCa	Acyl Carnitine	AcCa(14:1);AcCa(15:1)	2
AcHexChE	Acyl Hexosyl Cholesterol ester	AcHexChE(18:1);AcHexChE(17:1);AcHexChE(19:2)	3
AcHexCmE	Acyl Hexosyl Campesterol ester	AcHexCmE(18:3);AcHexCmE(18:2);AcHexCmE(16:0);AcHexCmE(16:1);AcHexCmE(18:0)	5
AcHexSiE	Acyl Hexosyl Sitosterol ester	AcHexSiE(16:0);AcHexSiE(18:3);AcHexSiE(18:2);AcHexSiE(18:0);AcHexSiE(16:1);AcHexSiE(15:0);AcHexSiE(18:1);AcHexSiE (15:1);AcHexSiE(20:1);AcHexSiE (20:0);AcHexSiE(17:0)	11

AcHexZyE	Acyl Hexosyl Zymosterol ester	AcHexZyE(22:0)	1
BisMeLPA	Bis-methyl lysophosphatidic acid	BisMeLPA(17:3);BisMeLPA(28:3)	2
BisMePA	Bis-methyl phosphatidic acid	BisMePA(36:5)	1
Cer	Ceramide	Cer(d18:2_20:0);Cer(d18:0_22:0);Cer(d18:2_18:2);Cer(t18:1_20:0);Cer(t22:0_18:2);Cer(t22:0_16:2);Cer(d18:0_24:0);Cer(t20:0_16:2);Cer(t18:1_22:0);Cer(t18:1_19:0);Cer(t18:1_24:0);Cer(d14:1_22:1);Cer(t16:0_22:1);Cer(t14:0_24:1);Cer(t18:1_18:0);Cer(m21:0_16:2);Cer(t16:0_18:2);Cer(t20:1_24:0);Cer(m17:0_20:0);Cer(m44:8);Cer(m17:0_21:0);Cer(t20:0_18:2);Cer(m17:0_23:0);Cer(m40:8);Cer(d19:0_16:2);Cer(m17:0_22:0);Cer(t44:2);Cer(t44:1);Cer(t20:1_22:0);Cer(m19:0_16:2);Cer(t38:3);Cer(m17:0_16:2);Cer(m18:1_15:0);Cer(m18:0_19:0);Cer(m17:0_24:0);Cer(m42:8);Cer(t17:0_19:2);Cer(m17:0_16:0);Cer(t39:2);Cer(m20:0_16:2);Cer(t19:0_18:2);Cer(d16:0_24:1);Cer(m17:0_18:0);Cer(t17:0_24:1);Cer(m21:0_16:3);Cer(t42:1);Cer(d19:0_16:0);Cer(d18:1_24:0);Cer(d18:0_15:1);Cer(t16:0_23:1);Cer(t18:1_21:0);Cer(t43:1);Cer(m18:1_17:0);Cer(t41:1);Cer(d18:2_21:0);Cer(d18:1_16:0);Cer(d18:0_20:0);Cer(d18:2_16:1);Cer(d18:2_18:1);Cer(t18:0_24:1);Cer(d18:2_16:0);Cer(d18:0_18:0);Cer(d18:0_16:0);Cer(d18:2_20:1);Cer(d18:2_22:1);Cer(d18:1_20:0);Cer(d18:2_24:1);Cer(d18:1_18:1);Cer(d18:1_17:2);Cer(d18:2_18:0);Cer(t18:1_16:0);Cer(d18:1_18:0);Cer(d18:2_22:0);Cer(d17:0_23:0);Cer(d18:3_29:4);Cer(t18:1_24:1);Cer(d18:2_19:0);Cer(d16:0_16:0);Cer(d20:0_16:0);Cer(d18:0_22:1);Cer(d12:0_16:0);Cer(d18:1_19:2);Cer(d16:0_18:0);Cer(d16:0_18:1);Cer(d14:0_18:0);Cer(d16:0_32:5);Cer(m18:0_20:0);Cer(d20:0_18:0);Cer(d39:1);Cer(d43:1);Cer(d47:5);Cer(d48:3);Cer(d16:0_12:0);Cer(d20:1_18:0);Cer(t18:1_20:4);Cer(d17:0_18:3);Cer(d39:6);Cer(m22:1);Cer(t26:3);Cer(t12:0_23:2);Cer(d44:5);Cer(d46:5);Cer(d14:0_16:0);Cer(d12:0_18:0);Cer(d17:0_20:0);Cer(d17:0_22:0);Cer(d48:6);Cer(d49:6);Cer(d49:5)	109
Ch-D7		Ch-D7	1
ChE	Cholesterol ester	ChE(21:3);ChE(20:3)	2
CmE	Campesterol ester	CmE(18:2);CmE(21:3)	2
Co	Coenzyme Q	Co(Q9);Co(Q10);Co(Q8);Co(Q6);Co(Q7)	5

DAP		DAP(O−11:0_O−5:0);DAP(O−15:0_O−5:0);DAP(O−12:0_O−4:0)	3
DG	Diglyceride	DG(16:0_18:2);DG(16:0_18:0);DG(18:2_18:3);DG(6:0_12:1);DG(18:3_18:3);DG(24:0_18:2);DG(18:4_22:6);DG(16:0_18:3);DG(18:1_18:3);DG(18:0_18:2);DG(18:0_18:0);DG(18:3_18:4);DG(20:0_18:3);DG(18:0_20:0);DG(16:0_18:1);DG(3:0_15:2);DG(18:1_18:2);DG(18:0_18:3);DG(18:2_18:2);DG(22:0_18:2);DG(16:0_19:1);DG(22:0_18:3);DG(14:0_18:3);DG(17:0_18:2);DG(16:0_16:0);DG(18:2_18:4);DG(16:0_21:1);DG(20:0_20:1);DG(18:2_22:6);DG(20:1_18:3);DG(15:2_18:2);DG(16:2_18:3);DG(16:0_15:2);DG(16:0_16:1);DG(18:2_24:6);DG(17:0_18:3);DG(18:3_20:3);DG(6:0_9:0);DG(6:0_10:0);DG(16:3_3:0);DG(28:6_3:0);DG(15:2_3:0);DG(18:3_3:0);DG(6:0_11:0);DG(5:0_14:2);DG(P−6:0_17:0);DG(16:2_20:2);DG(O−26:8_18:2);DG(19:3_3:0);DG(O−26:5_18:2);DG(16:0_24:7);DG(18:3_29:5);DG(12:1_18:4);DG(O−16:2_18:2);DG(O−18:4_18:2);DG(P−16:1);DG(O−15:1_24:0);DG(P−26:7);DG(O−15:1_16:0);DG(O−16:2_16:0);DG(O−16:2_17:0);DG(O−17:1_20:0);DG(17:2);DG(O−16:2_18:3);DG(O−15:2_22:0);DG(O−16:3_16:0);DG(O−18:4_16:0);DG(P−15:2_20:6);DG(P−22:7_13:1);DG(P−21:3);DG(O−17:1_14:0);DG(O−18:4_18:1);DG(15:0_23:1);DG(O−15:2_22:1);DG(16:2);DG(P−18:2);DG(O−15:1_21:0);DG(O−15:1_23:0);DG(O−21:1_18:2);DG(15:0_24:1);DG(15:0_25:1);DG(O−15:1_20:0);DG(13:1_3:0);DG(O−16:2_18:1);DG(O−30:6);DG(O−16:2_18:0);DG(O−17:1_23:0);DG(16:2_16:0);DG(O−18:4_20:1);DG(P−4:0_12:1);DG(O−17:1_17:3);DG(P−14:2_21:6);DG(O−27:6_20:3);DG(O−40:11);DG(P−6:0_12:0);DG(O−17:0);DG(O−26:6_20:3);DG(13:1_4:0);DG(3:0_16:0);DG(15:2_17:3);DG(O−37:8);DG(13:0_3:0);DG(18:2);DG(O−17:1_18:2);DG(O−42:12);DG(O−16:3_18:3);DG(P−18:4_18:3);DG(18:0_21:1);DG(O−26:8_18:1);DG(O−30:3_14:0);DG(O−16:2_14:0);DG(P−18:4_18:2);DG(O−18:4_14:0);DG(19:1_18:1);DG(13:0_4:0);DG(14:0_3:0);DG(18:3);DG(O−16:2_16:1);DG(P−18:4_16:0);DG(16:2_18:1);DG(P−18:4_18:1);DG(O−16:1_18:0);DG(O−16:3_20:1);DG(O−18:4_18:0);DG(O−15:1_21:3);DG(16:3_20:3);DG(21:4_17:1);DG(18:3_20:0);DG(22:1_16:0);DG(O−18:4_15:0);DG(O−18:4_18:3);DG(O−16:1_18:1);DG(15:0_20:1);DG(O−19:1_18:2);DG(16:0_20:0);DG(21:1_16:0);DG(20:2_18:2);DG(O−24:5_18:2);DG(15:2_29:6);DG(P−4:0_19:0);DG(6:0_19:2);DG(P−13:1_16:0);DG(O−22:6_21:4);DG(O−25:4_18:4);DG(P−28:8_18:1);DG(O−23:1);DG(15:2_17:0);DG(O−18:4_17:0);DG(O−16:2_20:0);DG(O−26:5_19:4);DG(O−45:8);DG(O−27:7_	171

		22:2);DG(O−13:0_13:1);DG(P−49:15);DG(15:1);DG(12:1_3:0);DG(P−20:3);DG(O−19:1);DG(O−26:5);DG(P−11:0_14:0);DG(O−14:1_16:0);DG(10:0_4:0);DG(O−18:0);DG(17:3_3:0);DG(18:4_3:0);DG(O−20:4_18:2);DG(15:0_26:1);DG(O−13:0_22:2);DG(P−18:4_20:0);DG(O−45:12);DG(O−25:1_20:3)	
DGDG	Digalactosyl diacylglycerol	DGDG(18:1_18:1);DGDG(18:0_18:1);DGDG(17:1_18:1);DGDG(16:0_18:0);DGDG(18:1_18:3);DGDG(18:1_16:1);DGDG(18:2_18:4);DGDG(18:2_17:1);DGDG(18:0_16:1);DGDG(18:1_22:1);DGDG(18:1_18:4);DGDG(18:1_26:1);DGDG(18:1_23:1);DGDG(18:1_20:2);DGDG(18:1_20:1);DGDG(18:1_15:1);DGDG(18:0_24:1);DGDG(26:1_16:1);DGDG(18:0_18:4);DGDG(6:0_22:3);DGDG(16:0_18:3);DGDG(16:0_18:2);DGDG(18:3_18:3);DGDG(16:0_18:1);DGDG(18:2_18:3);DGDG(18:0_18:2);DGDG(18:0_18:3);DGDG(18:1_18:2);DGDG(17:0_18:2);DGDG(16:0_16:0);DGDG(17:0_18:3);DGDG(24:0_18:2);DGDG(18:2_18:2);DGDG(22:0_18:2);DGDG(20:1_18:2);DGDG(8:0_22:1);DGDG(5:0_16:0);DGDG(20:3_18:2);DGDG(18:3_20:3)	39
DGMG	Digalactosyl monoacylglycerol	DGMG(18:3);DGMG(16:0);DGMG(18:2);DGMG(18:1)	4
FA	Fatty acid	FA(18:2);FA(23:0);FA(25:0);FA(18:4);FA(18:3);FA(24:0);FA(28:0);FA(16:0);FA(22:0);FA(20:2);FA(26:0);FA(28:1);FA(30:1);FA(20:1);FA(18:1);FA(18:0);FA(30:0);FA(27:0);FA(20:0);FA(16:1);FA(17:0);FA(10:0);FA(17:2);FA(21:0);FA(29:1);FA(29:0);FA(25:1)	27
Hex1Cer	Hexosyl ceramide	Hex1Cer(d16:1_20:1);Hex1Cer(d16:1_22:1);Hex1Cer(d16:0_18:2);Hex1Cer(d19:1_18:1);Hex1Cer(d16:2_22:1);Hex1Cer(d18:2_18:1);Hex1Cer(d18:2_20:1);Hex1Cer(d18:2_16:1);Hex1Cer(d18:2_19:1);Hex1Cer(d18:2_24:1);Hex1Cer(d18:2_22:1);Hex1Cer(d18:2_21:1);Hex1Cer(d18:2_18:0);Hex1Cer(d18:2_23:1);Hex1Cer(d18:2_16:0);Hex1Cer(d18:1_16:1);Hex1Cer(d18:1_20:1);Hex1Cer(d18:1_15:0)	18
LBPA		LBPA(16:0_18:3);LBPA(18:3_18:3);LBPA(16:0_18:2);LBPA(18:2_18:3);LBPA(18:1_18:3);LBPA(16:0_18:1);LBPA(2:0_18:2);LBPA(19:2);LBPA(20:2);LBPA(18:4_18:4);LBPA(16:2_16:0)	11
LPA	Lysophosphatidic acid	LPA(30:4);LPA(18:2)	2

LPC	Lyso phosphatidylcholine	LPC(18:3);LPC(18:2);LPC(16:0);LPC(18:1);LPC(16:1);LPC(16:2);LPC(P−18:4);LPC(22:2)	8
LPE	Lyso phosphatidylethanolamine	LPE(18:2);LPE(18:3)	2
LPEt	Lyso phosphatidylethanol	LPEt(18:2);LPEt(18:3);LPEt(O−15:2)	3
LPG	Lyso phosphatidylglycerol	LPG(16:0);LPG(18:3);LPG(18:2);LPG(16:1);LPG(18:1)	5
LPI	Lyso phosphatidylinositol	LPI(18:3);LPI(18:2)	2
LPIP2		LPIP2(14:2)	1
LPMt		LPMt(16:0);LPMt(18:3);LPMt(18:2);LPMt(18:1);LPMt(17:1);LPMt(18:0);LPMt(17:2);LPMt(16:1);LPMt(15:0);LPMt(17:0);LPMt(16:2);LPMt(19:2);LPMt(20:1);LPMt(17:3)	14
MG	Monoglyceride	MG(18:3);MG(16:0);MG(18:4);MG(22:1);MG(23:5);MG(18:0);MG(20:1);MG(17:3);MG(24:1);MG(18:2);MG(17:2);MG(P−18:4);MG(O−25:6);MG(25:8);MG(O−26:6);MG(P−16:2);MG(20:3);MG(19:4);MG(26:1);MG(28:1);MG(15:2);MG(O−18:4);MG(P−19:4);MG(P−15:2);MG(P−14:1);MG(14:0);MG(16:2);MG(P−17:3);MG(O−15:2);MG(O−21:3);MG(O−19:1);MG(O−21:6);MG(O−13:1);MG(O−26:7);MG(O−19:4);MG(O−20:5);MG(O−15:1)	37
MGDG	Monogalactosyl diacylglycerol	MGDG(16:0_16:0);MGDG(16:0_18:2);MGDG(18:3_18:3);MGDG(18:2_18:2);MGDG(18:0_18:2);MGDG(18:2_18:3);MGDG(16:0_18:3);MGDG(16:0_18:1);MGDG(17:0_18:3);MGDG(18:1_18:2);MGDG(18:0_18:3);MGDG(16:1_18:3);MGDG(3:0_15:2);MGDG(18:3)	14
MGMG	Monogalactosyl monoacylglycerol	MGMG(18:3);MGMG(16:1);MGMG(18:2);MGMG(16:0);MGMG(17:3);MGMG(18:1)	6
MePC	Methyl phosphatidylcholine	MePC(14:0);MePC(14:1)	2
OAHFA	O−Acyl−(gamma−hydroxy) FA	OAHFA(16:0_16:0);OAHFA(22:0_18:2);OAHFA(20:0_22:0);OAHFA(16:0_22:0);OAHFA(20:0_20:0);OAHFA(24:0_18:2);OAHFA(20:0_18:2);OAHFA(2:0_14:1);OAHFA(18:2_18:2);OAHFA(16:0_20:0);OAHFA(22:0_21:4);OAHFA(22:0_4:0)	12

PA	Phosphatidic acid	PA(16:0_18:2);PA(18:2_18:2);PA(16:0_18:1);PA(18:2_18:3);PA(16:0_18:3);PA(17:0_18:2);PA(18:3_18:3);PA(18:1_18:2);PA(18:0_18:2);PA(19:3_18:3);PA(17:0_16:0);PA(19:2_18:2);PA(17:1_2:0);PA(17:2_2:0);PA(22:2);PA(21:2_2:0);PA(47:13);PA(17:0_18:3);PA(37:3);PA(O−5:0_18:2)	20
PC	Phosphatidylcholine	PC(16:0_18:1);PC(16:0_18:3);PC(16:0_18:2);PC(18:3_18:3);PC(18:2_18:3);PC(18:0_18:2);PC(18:0_18:1);PC(18:1_18:3);PC(17:0_18:3);PC(20:0_18:3);PC(16:3_18:2);PC(18:2_18:2);PC(18:1_18:2);PC(18:1_16:0);PC(18:2_16:0);PC(18:3_18:2);PC(35:3);PC(36:1);PC(34:3);PC(38:4);PC(16:1_18:1);PC(34:1);PC(35:2);PC(36:4);PC(36:2);PC(19:2_17:0);PC(18:2);PC(34:4);PC(32:1);PC(38:3);PC(18:3_16:0);PC(34:2);PC(14:0_22:4);PC(36:3);PC(33:2);PC(14:1_18:0);PC(14:1_16:0);PC(15:1_16:0);PC(16:1_18:0);PC(36:5);PC(21:2);PC(34:0);PC(16:1_16:0);PC(19:2_18:1);PC(O−23:6_16:0);PC(30:2);PC(16:0_16:0);PC(18:4_17:3);PC(38:9);PC(O−18:1);PC(33:3);PC(16:2_18:3);PC(37:4);PC(38:5)	54
PE	Phosphatidylethanolamine	PE(16:0_18:3);PE(18:1_18:2);PE(16:0_18:1);PE(18:2_18:3);PE(18:2_18:2);PE(18:3_18:3);PE(16:0_18:2);PE(17:2_16:0);PE(17:1_16:0);PE(17:2_18:3);PE(17:2_18:2);PE(19:2_18:2);PE(19:1_16:0);PE(O−29:2_18:2);PE(19:2_16:0);PE(17:1_18:2);PE(19:0_16:0);PE(19:2_18:3);PE(17:0_16:0);PE(19:1_15:0);PE(19:1_17:0);PE(17:2_18:0);PE(19:1_18:2);PE(17:2_17:0);PE(17:1_18:0);PE(17:1_18:1);PE(17:2_21:4);PE(19:1_14:0);PE(20:5_18:2);PE(17:3_16:0);PE(17:1_17:0);PE(O−11:0_15:2);PE(O−5:0_12:1);PE(17:2_15:0);PE(17:1_24:0);PE(17:2_14:0)	36
PEt	Phosphatidylethanol	PEt(16:0_16:1);PEt(19:3_16:0);PEt(16:3_16:0);PEt(15:0_20:5)	4
PG	Phosphatidylglycerol	PG(16:0_16:0);PG(16:0_18:2);PG(18:3_18:3);PG(16:0_18:1);PG(P−16:3_18:2);PG(O−16:3_16:0);PG(16:2_18:2);PG(P−16:3_16:0);PG(O−16:3_18:2);PG(19:3_16:0);PG(P−16:3_18:3);PG(19:3_18:2);PG(O−19:4_18:2);PG(16:3_16:0);PG(16:2_16:0);PG(P−15:2_18:2);PG(O−16:3_18:3);PG(O−19:4_18:3);PG(19:3_18:3);PG(16:0_18:3);PG(P−19:4_18:2);PG(16:0_24:0);PG(28:0_16:0);PG(P−19:4_19:2);PG(25:8_18:2);PG(28:1_16:0);PG(28:0_18:2);PG(19:0_18:3);PG(28:1_18:3);PG(30:0_18:2);PG(28:0_18:3);PG(P−15:2_17:0);PG(P−15:2_19:2);PG(23:7_18:3);PG(24:2_18:0);PG(17:2_18:3);PG(O−19:4_17:0);PG(O−19:4_19:2);PG(P−19:4_19:0);PG(P−23:7_16:0);PG(O−19:4_19:3);PG(18:1_18:3)	42

PI	Phosphatidylinositol	PI(16:0_18:2);PI(16:0_18:3);PI(18:3_18:3);PI(26:1_14:2);PI(12:1_18:3);PI(26:5_16:0);PI(24:3_18:3);PI(26:6_18:2);PI(28:5_18:2);PI(21:0_3:0);PI(24:4_16:0);PI(26:7_18:3);PI(26:5_18:1)	13
PMt		PMt(15:0_18:2);PMt(16:0_18:1);PMt(18:2_18:3);PMt(18:1_18:2);PMt(18:0_18:2);PMt(16:0_18:3);PMt(16:0_18:2);PMt(17:0_18:2);PMt(18:0_18:1);PMt(14:0_18:2);PMt(20:0_18:2);PMt(17:0_18:1);PMt(18:0_18:3);PMt(18:2_20:3);PMt(23:0_18:2);PMt(22:0_18:2);PMt(18:2_20:2);PMt(24:0_18:2);PMt(19:0_18:2);PMt(14:0_18:3);PMt(16:1_18:3);PMt(17:2_18:2);PMt(16:0_16:1);PMt(17:1_18:2);PMt(20:1_18:2);PMt(15:1_18:2);PMt(21:0_18:2);PMt(16:0_18:0);PMt(20:0_18:3);PMt(19:1_18:2);PMt(25:0_18:2);PMt(15:0_16:0);PMt(16:0_17:0);PMt(24:0_18:3);PMt(17:3_18:3);PMt(18:1_18:3);PMt(18:3_20:3)	37
PS	Phosphatidylserine	PS(18:2_18:2);PS(20:2_16:0);PS(P−18:4_17:3)	3
SPH	Sphingosine bases	SPH(d14:0);SPH(d20:0);SPH(d16:0);SPH(d18:2);SPH(d18:1);SPH(d18:0);SPH(d16:1);SPH(t18:1);SPH(d15:0);SPH(d19:0);SPH(t20:1);SPH(d17:0)	12
SQDG	Sulfoquinovosyl diacylglycerol	SQDG(16:0_16:0);SQDG(16:0_18:2);SQDG(18:0_18:3);SQDG(18:0_18:2);SQDG(17:0_18:3);SQDG(18:3_20:5);SQDG(16:0_18:1);SQDG(18:2_18:2);SQDG(16:0_17:0);SQDG(20:5_18:4);SQDG(18:0_16:1);SQDG(17:3_16:1);SQDG(18:2_20:5);SQDG(34:0);SQDG(33:2)	15
SQMG	Sulfoquinovosyl monoacylglycerol	SQMG(16:0);SQMG(18:2);SQMG(18:3)	3
SiE	Sitosterol ester	SiE(18:2);SiE(17:2);SiE(18:4);SiE(24:6)	4
TG	Triglyceride	TG(16:0_16:0_18:2);TG(16:0_16:0_18:1);TG(16:0_16:0_18:0);TG(16:0_18:2_18:3);TG(14:0_18:2_18:3);TG(16:0_18:0_18:0);TG(16:0_20:0_18:2);TG(18:3_18:3_18:3);TG(14:0_16:0_18:2);TG(16:0_16:0_18:3);TG(16:0_20:0_18:1);TG(16:0_18:0_18:2);TG(18:0_18:1_18:3);TG(20:0_18:2_18:3);TG(18:0_20:0_18:1);TG(20:0_20:0_18:2);TG(14:0_16:0_18:3);TG(14:0_16:0_18:1);TG(15:0_16:0_18:2);TG(20:0_18:1_18:2);TG(16:0_20:0_20:0);TG(20:0_20:0_18:1);TG(19:1_18:2_18:3);TG(20:0_22:0_18:2);TG(16:0_17:0_18:2);TG(16:1_18:2_18:3);TG(18:2_18:2_18:3);TG(16:0_19:0_18:2);TG(18:1_18:2_18:2);TG(19:0_20:0_18:2);TG(22:0_18:3_18:3);TG(16:0_18:1_18:3);TG(18:0_18:2_18:3);TG(16:0_17:0_18:3);TG(16:0_18:1_18:2);TG(18:1_18:2_18:3);T	269

		G(19:0_18:2_18:2);TG(18:0_20:0_18:2);TG(22:0_18:2_18:2);TG(22:0_18:2_18:3);TG(24:0_18:2_18:2);TG(18:0_18:1_18:2);TG(6:0_16:0_12:1);TG(16:0_18:0_18:3);TG(16:0_17:0_18:1);TG(17:0_18:2_18:3);TG(20:0_21:0_18:3);TG(20:0_22:0_18:3);TG(18:2_17:3_18:3);TG(17:2_18:2_18:3);TG(24:0_18:2_18:3);TG(20:0_18:2_18:2);TG(19:0_18:2_18:3);TG(3:0_18:2_18:2);TG(18:0_18:3_18:3);TG(20:0_18:1_20:1);TG(16:0_16:1_18:2);TG(20:0_20:0_18:3);TG(15:0_16:0_18:1);TG(20:0_21:0_18:2);TG(15:0_18:2_18:3);TG(17:0_18:1_18:3);TG(16:0_18:2_18:4);TG(16:0_18:0_18:1);TG(18:2_20:2_18:3);TG(22:1_18:2_18:3);TG(23:0_18:2_18:2);TG(14:0_18:3_18:3);TG(19:2_18:3_18:3);TG(3:0_16:0_15:2);TG(3:0_15:2_18:3);TG(15:0_16:0_17:0);TG(16:0_18:3_18:3);TG(16:0_18:1_18:4);TG(17:0_18:1_18:2);TG(20:1_18:3_18:3);TG(20:1_18:2_18:3);TG(16:0_22:0_18:2);TG(18:1_18:1_22:1);TG(17:2_18:2_18:2);TG(18:1_24:1_18:3);TG(2:0_16:0_18:3);TG(15:0_16:0_18:3);TG(18:1_20:1_18:2);TG(14:0_16:0_16:0);TG(3:0_16:0_18:2);TG(O−14:2_4:0_18:2);TG(3:0_16:0_18:3);TG(3:0_19:2_18:3);TG(18:1_3:0);TG(3:0_6:0_9:0);TG(18:2_16:0);TG(O−15:1_18:0_3:0);TG(3:0_16:0_19:2);TG(3:0_18:2_19:2);TG(14:2_4:0_18:0);TG(3:0_18:2_18:3);TG(3:0_4:0_14:1);TG(6:0_12:1_22:6);TG(O−15:1_3:0_20:1);TG(6:0_12:1_24:8);TG(O−15:2_6:0_15:1);TG(O−17:0_20:0_3:0);TG(O−15:2_3:0_4:0);TG(3:0_18:3_19:3);TG(4:0_5:0_9:0);TG(O−5:0_3:0_21:5);TG(O−15:1_3:0_18:3);TG(12:1_6:0_18:3);TG(16:0_16:0_18:4);TG(18:2_16:0_18:4);TG(18:2_16:0_18:3);TG(18:1_18:2_18:4);TG(18:1_18:3_18:3);TG(18:1_20:2_18:3);TG(O−5:0_3:0_23:6);TG(O−28:6_3:0);TG(O−30:8_3:0_3:0);TG(O−15:0_3:0_18:0);TG(12:1_6:0_18:1);TG(36:8_16:0);TG(16:1_18:1_18:2);TG(36:8_18:3);TG(3:0_16:0_18:0);TG(18:2_18:1);TG(7:0_3:0_21:6);TG(O−15:2_3:0_18:3);TG(O−17:3_3:0_2:0);TG(4:0_5:0_11:0);TG(3:0_16:0_19:3);TG(14:1_4:0_16:0);TG(26:7);TG(O−18:2_24:8);TG(O−18:0_24:8);TG(O−15:1_25:1);TG(9:0_16:0);TG(O−15:2_20:0_3:0);TG(O−10:0_3:0_7:0);TG(47:13_16:0);TG(17:1_4:0_7:0);TG(14:1_4:0);TG(O−10:0_3:0_6:0);TG(O−13:0_3:0_18:2);TG(O−14:1_16:0_10:0);TG(15:0_22:2_21:4);TG(O−28:0_3:0_3:0);TG(O−14:1_16:0_8:0);TG(P−11:0_3:0_5:0);TG(O−8:0_3:0_8:0);TG(O−15:2_16:1_3:0);TG(O−14:1_16:0_9:0);TG(O−15:1_16:0_10:0);TG(17:0_19:3_20:4);TG(21:3_19:1_18:2);TG(15:0_22:1_21:4);TG(42:2_18:0);TG(O−13:0_3:0_18:0);TG(12:1_22:1_24:2);TG(15:2_24:2_21:4);TG(47:11_16:0);TG(O−16:2_3:0_7:0);TG(15:1_4:0_7:0);TG(O−12:1_3:0_5:0);TG(3:0_6:0_14:1);TG(O−13:0_15:2_3:0);TG(P−15:2_3:0_18:1);TG(18:1_1	

		8:0);TG(4:0_18:4_18:4);TG(18:1_18:1_18:4);TG(20:2_18:1_18:3);TG(16:1_18:1_22:4);TG(18:2_20:0_18:3);TG(20:1_18:1_18:3);TG(20:0_19:1_18:3);TG(18:2_18:0);TG(18:3_22:0_18:1);TG(2:0_4:0_22:7);TG(O−15:2_19:0_6:0);TG(24:7_21:5);TG(O−8:0_6:0_22:2);TG(O−16:0_24:8);TG(42:2_18:3);TG(O−14:0_21:0_2:0);TG(P−8:0_16:0);TG(O−25:5_28:3);TG(O−14:2_4:0_24:7);TG(15:0_24:2_21:4);TG(3:0_16:0_16:0);TG(O−14:0_21:0_6:0);TG(P−41:12_9:0);TG(37:2_22:5);TG(36:5_23:1);TG(4:0_21:3_21:3);TG(4:0_9:0_9:0);TG(P−8:0_4:0_20:0);TG(O−18:4_17:1_3:0);TG(24:7_18:4);TG(49:15_18:3);TG(O−15:2_14:0_3:0);TG(O−14:1_16:0_5:0);TG(O−14:1_16:0_7:0);TG(O−14:2_4:0);TG(18:1);TG(7:0_5:0_6:0);TG(O−17:3_4:0);TG(14:2_4:0_16:0);TG(O−15:1_19:2);TG(O−13:0_18:2_3:0);TG(O−15:1_3:0_18:0);TG(18:2_18:3);TG(O−15:1_16:0_9:0);TG(O−14:1_16:0_11:0);TG(P−26:8_18:2);TG(16:2_18:0_18:1);TG(18:4_18:2_18:2);TG(40:4_18:1);TG(18:2_22:0_18:3);TG(35:3_23:0);TG(34:1_24:2);TG(20:2_18:0_20:1);TG(36:2_23:7);TG(47:13_18:3);TG(O−9:0_3:0_8:0);TG(O−12:0_3:0_5:0);TG(22:5_3:0);TG(O−10:0_6:0_18:0);TG(O−25:0_4:0_5:0);TG(3:0_14:2_21:4);TG(21:1_19:3);TG(15:2_6:0_20:3);TG(O−22:6_22:7);TG(P−18:4_24:8_3:0);TG(24:7_21:4);TG(16:2_18:2_18:3);TG(O−22:4_18:3_18:4);TG(42:1_18:2);TG(47:13_18:2);TG(P−5:0_3:0_20:4);TG(P−10:0_16:0);TG(O−8:0_3:0_18:3);TG(P−15:2_16:0_3:0);TG(P−7:0_18:2_9:0);TG(P−4:0_18:4_14:2);TG(O−14:2_3:0_18:2);TG(18:2_20:3);TG(P−15:2_4:0_24:5);TG(O−38:8_5:0);TG(3:0_7:0_16:1);TG(O−14:0_4:0_27:8);TG(28:2_6:0_24:6);TG(22:4_18:2_18:2);TG(47:12_16:0);TG(P−4:0_3:0_15:2);TG(P−8:0_10:0);TG(O−11:0_3:0_4:0);TG(22:4_5:0_5:0);TG(P−19:4_24:8_3:0);TG(P−19:4_24:7_3:0);TG(O−25:5_30:3);TG(P−4:0_16:0_18:0);TG(34:2_24:5);TG(4:0_14:2);TG(6:0_3:0_11:0);TG(P−4:0_16:0_12:1);TG(O−10:0_21:5_3:0);TG(3:0_13:0_18:3);TG(15:0_19:3);TG(O−7:0_6:0_22:2);TG(P−16:3_5:0_15:1)	
WE	Wax esters (fatty acid esters)	WE(O−22:0_23:6);WE(O−25:2_23:1);WE(O−25:2_24:1);WE(O−11:0_16:2);WE(O−27:5);WE(O−34:2);WE(O−37:0);WE(O−28:6_18:3);WE(O−28:6_18:2);WE(O−35:0);WE(O−29:6_18:3);WE(O−29:6_18:2);WE(O−29:6_18:1);WE(O−6:0_21:2);WE(O−31:4);WE(O−29:5_18:3);WE(O−29:1);WE(O−22:3);WE(O−20:5_15:0);WE(O−20:5_19:1);WE(O−26:2);WE(O−21:0);WE(O−24:3);WE(O−38:9);WE(O−10:0_17:2);WE(O−9:0_18:2);WE(O−5:0_22:2);WE(O−25:5_19:2);WE(O−18:1);WE(O−39:0	42

		);WE(O−28:5_17:1);WE(O−29:3_16:1);WE(O−32:2);WE(O−28:5_18:2);WE(O−30:6_18:1);WE(O−4:0_18:4);WE(O−20:2);<br>WE(O−15:2_13:1);WE(O−6:0_14:0);WE(O−30:3);WE(O−34:3);WE(O−25:2_18:2)	
d5−DG		d5−DG(34:5)	1
d5−TG	Deuterated triglyceride	d5−TG(38:5_18:3);d5−TG(36:6_20:2);d5−TG(40:5_18:3);d5−TG(56:9);d5−TG(34:5_18:3)	5

Table S8. UHPLC–MS Analysis of Total Lipids in *ms*-N125* and Fertile Sibling (Wild Type = WT).

Subclass	Group	n	sum	se
AEA	*ms*-N125*	4	1285250.485	94597.38188
AEA	WT	4	788708.9504	42229.08197
AcCa	*ms*-N125*	4	2550568.672	412937.1196
AcCa	WT	4	1494746.329	293437.5927
AcHexChE	*ms*-N125*	4	3492699.398	196157.4087
AcHexChE	WT	4	2115908.007	241271.4377
AcHexCmE	*ms*-N125*	4	4605422.262	255916.7536
AcHexCmE	WT	4	3700166.627	335876.2044
AcHexSiE	*ms*-N125*	4	22939187.22	2706505.474
AcHexSiE	WT	4	8889044.642	768528.5158
AcHexZyE	*ms*-N125*	4	3542404.101	1561101.107
AcHexZyE	WT	4	1067833.626	415101.8256
BisMeLPA	*ms*-N125*	4	789263.2719	143500.3072
BisMeLPA	WT	4	19789200.69	2612040.305
BisMePA	*ms*-N125*	4	111781.4361	68933.58024
BisMePA	WT	4	319071.8842	49431.6227
Cer	*ms*-N125*	4	137740802.6	7526875.29
Cer	WT	4	162247388.3	17702897.63
ChE	*ms*-N125*	4	1013606.826	319498.1165
ChE	WT	4	1219158.107	240586.4624
CmE	*ms*-N125*	4	2106059.037	164223.7015
CmE	WT	4	6035410.948	675015.7287
Co	*ms*-N125*	4	10092911.56	1321044.224
Co	WT	4	22616133.99	1482570.209
D5TG	*ms*-N125*	4	10903379.32	615810.0786
D5TG	WT	4	101934590	23986469.99
DG	*ms*-N125*	4	395587808.9	26084123.44
DG	WT	4	689739789.4	25339673.31
DGDG	*ms*-N125*	4	115310352.4	10101352.86
DGDG	WT	4	109239092.8	11588003.66
DGMG	*ms*-N125*	4	1224193.614	132786.8047
DGMG	WT	4	1579272.258	914710.5228
FA	*ms*-N125*	4	23625115.32	3261449.896
FA	WT	4	17353080.18	10034408.67
Hex1Cer	*ms*-N125*	4	35870486.11	917272.2582
Hex1Cer	WT	4	41949721.94	2048542.157
LPA	*ms*-N125*	4	379947.5555	114355.3095
LPA	WT	4	114475.6781	67763.99678
LPC	*ms*-N125*	4	3432309.942	322617.3028
LPC	WT	4	41772694.79	3539185.952

LPE	*ms*-N125*	4	71881.93297	6258.968418
LPE	WT	4	1270365.051	771490.4918
LPEt	*ms*-N125*	4	145734.372	13404.39719
LPEt	WT	4	1027104.959	170376.5963
LPG	*ms*-N125*	4	2825468.99	909826.4977
LPG	WT	4	2529815.843	1484108.283
LPI	*ms*-N125*	4	19357.27211	12231.78328
LPI	WT	4	83412.78512	50881.48808
MG	*ms*-N125*	4	66158283.17	5106695.119
MG	WT	4	119474747	8018636.216
MGDG	*ms*-N125*	4	110488236.5	14061123.63
MGDG	WT	4	158020943.9	10082190.59
MGMG	*ms*-N125*	4	10163559.9	1604559.16
MGMG	WT	4	20490568.23	11845121.78
MePC	*ms*-N125*	4	443039.4655	56577.66542
MePC	WT	4	1964155.63	290678.9783
OAHFA	*ms*-N125*	4	814093.3988	27088.71849
OAHFA	WT	4	136072.401	84940.52042
PA	*ms*-N125*	4	65601404.35	3624105.753
PA	WT	4	61335387.59	38059154.32
PC	*ms*-N125*	4	47205565.96	3642102.537
PC	WT	4	116549870.5	15362237.41
PE	*ms*-N125*	4	223593813.2	11580454.3
PE	WT	4	655081053.1	46381427.11
PEt	*ms*-N125*	4	1251695.443	113236.4016
PEt	WT	4	1222327.684	147050.0849
PG	*ms*-N125*	4	19398702.23	681676.9133
PG	WT	4	12582678.5	1955386.471
PI	*ms*-N125*	4	27428553.19	2251148.282
PI	WT	4	19454715.94	2492728.919
PS	*ms*-N125*	4	397884.1507	64204.9133
PS	WT	4	307924.2433	70041.169
SPH	*ms*-N125*	4	75264195.39	24906819.79
SPH	WT	4	25473163.23	22826891.37
SQDG	*ms*-N125*	4	28245369.03	3408394.801
SQDG	WT	4	23828925.9	13971768.61
SQMG	*ms*-N125*	4	266992.3608	72034.39313
SQMG	WT	4	538049.563	310841.4073
SiE	*ms*-N125*	4	15284504.01	950204.6291
SiE	WT	4	65370975.06	2606025.616
TG	*ms*-N125*	4	980255585.4	45669853.48
TG	WT	4	1212357060	103878364.7

WE	*ms*-N125*	4	20677495.62	781058.3708
WE	WT	4	53259361.75	2627440.634

Table S9. Carbon chain length analysis of lipid molecules in mutant and WT.

	**Chain Length (n=4)**	**Relative level of Wild Type**	**Relative level of *ms*-N125***	**se (Wild Type)**	**se (*ms*-N125*)**
DG	25	217119.3129	238156.7213	36826.17904	25137.88903
	26	531743.9428	914115.6915	18752.7324	109352.718
	29	142920.6683	47514.58221	20308.91142	3016.422113
	30	1149335.609	3169260.418	50080.02387	362125.2553
	31	2726524.287	3892009.961	123757.6325	388133.5849
	32	111067175.2	27539145.01	7840685.245	1381578.067
	33	841444.635	3454082.138	33874.79218	130852.4629
	34	200766660.2	51975671.35	6728919.302	1628171.278
	35	27074930.54	14149387.34	1635015.358	377108.797
	36	248667994.7	166237527.4	10695604.26	1911432.41
	37	8001623.506	15481172.63	289259.2409	702790.0593
	38	25516794.12	14573661.27	882572.9282	413895.7119
	39	12551876.87	21186177.88	900528.1998	1343402.43
	40	8659872.626	12689160.24	173510.6406	245344.6777
	41	589962.5038	1510556.488	65300.89737	48274.92318
	42	1479536.726	1529646.691	58643.52983	40935.99042
	43	1326987.805	9771.677743	143023.6223	2935.88674
	44	1191225.293	517792.2363	92562.78925	96872.50724
	45	3346571.905	386849.8793	121609.6454	10086.40947
	46	5193174.577	5900.461779	244141.991	2138.994068
	47	3947152.698	5798.023375	162890.1759	931.3021828
	49	2102781.328	179370.0359	77742.26056	15860.18179
MGDG	16	323250.4504	278358.8863	99675.82302	9548.235266
	17	63468.9435	279089.0789	18576.53453	28577.87588
	18	20849415.97	9875847.945	5782601.497	766617.9537
	32	82388.9154	114023.0012	23947.43091	3362.034948
	34	10657350.99	13917062.85	343479.0003	1180680.045
	35	486581.9109	1226342.156	25748.30788	86923.99182
	36	146049055	94961072.5	4893478.548	6598244.194
PC	17	16336601.45	2531091.966	1517324.295	125161.0825
	18	843331.6716	29720.64731	50611.12027	3460.104382
	21	143089.1755	0	6857.724434	0
	26	0	7184.772584	0	2465.920385
	30	285388.2288	649157.1422	19209.68026	51385.14153
	31	547564.9013	2261815.414	27548.51114	124120.7831
	32	26048727.62	31116029	2731024.488	1872065.562

	33	175525411.9	88428243.75	9674020.485	2529574.575
	34	74625207.34	14973928.22	6260035.861	566912.3646
	35	185286504.6	108307468.2	2215277.722	2826648.873
	36	17042869.74	5351023.678	1925050.299	108300.0028
	37	270254132.5	15559223.96	14197356.47	396226.6956
	38	2949086.095	1132623.292	182641.2446	34059.85308
	39	314.7013871	36170.50328	154.5717559	18080.58852
	41	42618.89051	132675.5493	10556.55429	15663.36711
	47	1700074.819	283023.037	88011.96806	16743.22923
PE	17	16336601.45	2531091.966	1517324.295	125161.0825
	26	0	7184.772584	0	2465.920385
	31	175925.665	38457.87021	16142.14206	4260.124103
	32	110958.7277	416175.8851	9927.709021	16595.88373
	33	174835012.3	88200178.57	9631676.853	2529538.899
	34	5860590.305	5509718.435	733285.0185	270176.7929
	35	183305497.2	107950335.4	2091007.756	2850872.261
	36	2492587.24	3125812.759	206263.5652	69990.87922
	37	269893993	15362730.74	14203180.7	476114.0261
	38	327193.489	36428.22268	55028.21879	3046.137039
	41	42618.89051	132675.5493	10556.55429	15663.36711
	47	1700074.819	283023.037	88011.96806	16743.22923

Table S10. Carbon chain saturation analysis of lipid molecules in mutant and WT.

	Chain Saturation (n=4)	Relative level of Wild Type	Relative level of *ms*-N125*	se (Wild Type)	se (*ms*-N125)*
DG	0	18639917.26	31661317.32	3287952.075	3191336.472
	1	56410173.35	84978521.76	3484779.985	5515156.527
	2	208871919.4	58857603.91	8876823.117	1905535.199
	3	27238786.4	12922612.88	1143049.683	410111.1617
	4	123074444	59909466.14	5290925.443	2559148.948
	5	152450980.2	46604969.08	6831176.095	834551.5416
	6	69068280.78	82132052.16	322314.1616	659348.4337
	7	7345877.167	4787835.767	288042.6944	138549.5394
	8	10917928.36	12110026.87	510782.1199	293463.6526
	9	12232714.67	22677.92603	522408.9406	4968.761965
	10	1415340.552	381882.1542	243184.1032	41304.86005
	11	756328.5023	393918.9718	18640.08475	19881.22315
	12	1260067.436	647538.9981	52367.7701	23962.82104
	15	57031.30292	177384.9372	12888.11574	15870.0461
MGDG	0	82388.9154	114023.0012	23947.43091	3362.034948
	1	529973.8491	511116.832	68577.92273	50902.63264
	2	8740499.118	10806830.36	227029.4503	1108190.582

	3	7198551.001	6116600.045	253866.5967	416694.528
	4	26290185.81	4047617.248	1318953.306	166647.0305
	5	9073646.648	5820711.235	805885.3716	314561.903
	6	106105698.6	83071337.8	6297626.758	6136073.006
PC	0	3517099.797	433476.2853	319331.8397	46421.12205
	1	32687447.11	35521666.71	3217272.834	1765987.891
	2	24608915.42	2383560.353	1843364.522	85654.89834
	3	18552698.95	1143211.672	2951647.322	88197.69635
	4	23931423.63	6557738.814	309328.4646	206743.2602
	5	7379292.513	893566.1414	666359.7583	115687.4818
	6	596514.3923	36170.50328	258298.3015	18080.58852
	7	48380.85982	236175.4779	8513.590536	19556.26365
	9	910976.8507	0	171198.1319	0
PE	0	7136585.544	2313605.097	336944.7189	123997.7827
	1	80145271.36	97634014.89	3412800.179	2829822.862
	2	139195386.3	27317256.57	11965704.03	1187074.948
	3	283371490.8	72417561.26	9743392.662	2261248.33
	4	82956991.65	21961379.72	1411908.142	892959.9447
	5	60522449.11	1913567.46	7248820.851	193638.7619
	6	1752777.287	35660.50748	290231.0404	3205.064502
	7	101.0747982	767.7151969	20.78992855	241.2402571

Table S11. The relative levels of lipids which showed significant difference between *ms*-N125* and Wild Type.

	ms-N125*				Wild Type			
**ID**	**M1**	**M2**	**M3**	**M4**	**W1**	**W2**	**W3**	**W4**
PE(19:1_18:2)	-0.913058688	-0.891647024	-0.91083764	-0.892149268	0.563402189	1.076481459	0.575637747	1.392171225
TG(16:0_18:1_18:3)	0.610383364	0.835533746	0.996214425	0.584696955	-0.322556761	-0.835492127	0.077228517	-1.946008118
TG(18:1_18:2_18:3)	0.480059208	0.684853619	1.288994201	0.625532193	3.57497E-05	-1.561089192	-0.185618591	-1.332767187
PMt(16:0_18:2)	0.309752289	1.004494556	0.78012632	0.876043978	0.180205529	-0.16412543	-1.493248621	-1.493248621
DG(O−18:4_18:2)	0.277259331	1.359566177	0.085827216	1.127555545	0.242664036	-1.369704	-0.427804329	-1.295363976
PE(17:1_18:2)	0.440380618	1.376561806	0.723395331	0.28850253	0.304888429	-1.115603107	-0.326909865	-1.691215741
TG(18:0_18:2_18:3)	0.60574871	0.727434588	1.317163557	0.612702172	-1.31243203	-1.368287109	0.047466224	-0.629796114
PE(17:1_16:0)	0.033405745	1.655770369	0.298541324	0.996140112	-0.20466056	-1.537050864	-0.618654679	-0.623491445
PE(17:2_18:3)	-0.803439874	-0.808553161	-0.764859927	-0.784767172	-0.065328168	1.619129578	0.264712253	1.343106472
DG(O−16:2_18:3)	-0.836131355	-0.840451285	-0.788370529	-0.816529031	-0.170154211	1.517823972	0.739177322	1.194635118
DG(18:2_18:2)	-0.929345341	-0.633000186	-0.644426367	-0.699357066	1.797178538	0.053478567	1.249373399	-0.193901543
TG(O−14:2_4:0_18:2)	-1.014077318	-0.739672899	-0.753391771	-0.794665447	1.740226901	0.542985941	0.947160283	0.07143431
SiE(18:4)	-0.92981731	-0.93284729	-0.918919963	-0.926652557	0.64159968	0.924580867	1.097802684	1.044253888
TG(16:0_16:0_18:1)	-0.975444448	-0.890813969	-0.630123079	-0.917238367	0.282794737	1.014019618	1.636302337	0.480503173
DG(O−16:2_18:2)	-1.044582848	-0.949944633	-0.658700837	-0.928214164	0.819199937	0.39377704	0.984207476	1.38425803
MGDG(18:2_18:2)	-0.84786472	-0.963832907	-0.859218696	-0.915400358	1.184713279	0.347048989	0.761428109	1.293126305
TG(O−13:0_3:0_18:2)	-0.890977435	-0.863690235	-0.878077284	-0.881571289	0.688266346	1.134666086	1.445876646	0.245507164
WE(O−28:5_17:1)	-0.932822251	-0.938699068	-0.935026497	-0.916209343	0.732629603	1.105651923	0.950486521	0.933989112
LPC(16:2)	-0.928232074	-0.928332315	-0.911147353	-0.928296094	0.739206099	1.209566441	1.029772973	0.717462323
BisMeLPA(17:3)	-0.897741889	-0.894463543	-0.873927722	-0.897415774	0.420681402	1.326167341	1.254035857	0.562664329
Co(Q9)	-0.441200447	-1.185820743	-1.197782926	-0.628192934	0.996569748	0.217632568	1.011589896	1.227204838
MG(18:3)	-0.899023018	-1.302660193	-0.328199437	-0.974318648	1.036128504	1.293983588	0.569383845	0.604705359
PE(19:2_18:2)	-1.060962507	-1.018499725	-0.745076382	-0.763655087	0.355071607	1.308908503	1.025717602	0.898495991
DG(O−18:4_16:0)	-1.047842539	-0.862137205	-0.897032927	-0.669002083	0.454717044	1.111727511	1.520726251	0.388843949
PC(34:4)	-1.049854033	-0.970902723	-0.839083193	-0.863326501	0.943896493	0.829230842	0.861286293	1.088752821
DG(18:0_18:3)	-0.817952426	-0.737962004	-0.68185911	-0.746096333	-0.24866949	1.866564854	1.061094564	0.304879946
PE(19:2_16:0)	-0.722610733	-0.686773794	-0.70274388	-0.708793993	-0.711081089	0.726888442	1.5467783	1.258336746
PE(19:2_18:3)	-0.843157925	-0.84387399	-0.806259825	-0.824270683	-0.197372395	1.286587312	0.9264835	1.301864005

DG(O−13:0_22:2)	-0.825273279	-0.824124969	-0.81670056	-0.827228377	-0.230660383	1.149259078	0.969686599	1.405041892
DG(16:0_18:1)	-0.917055779	-0.779352466	-0.874738239	-0.802878696	0.095158797	1.207338665	1.550303388	0.52122433
WE(O−25:5_19:2)	-0.832142729	-0.832770488	-0.832770488	-0.816921015	-0.015585143	0.584184699	1.638876914	1.10712825
PE(O−5:0_12:1)	-0.88461491	-0.794403824	-0.753891517	-0.859034136	1.779840042	0.322739115	0.982497662	0.206867568
DGDG(18:2_18:3)	-0.944007766	-0.950186	-0.861463705	-0.914949953	1.334982704	0.909286468	0.696712119	0.729626133
LPC(16:1)	-0.994234598	-0.927288494	-0.900462106	-0.856367546	1.31152412	0.839515159	0.853689516	0.67362395
TG(20:0_20:0_18:2)	-0.95479586	-0.9309506	-0.883859325	-0.898001582	0.710912266	1.225749523	1.118590603	0.612354976
TG(O−18:4_17:1_3:0)	-0.912014159	-0.87935795	-0.896415193	-0.87502368	0.592347582	1.193702929	1.373917002	0.40284347
DG(21:4_17:1)	-0.912746069	-0.896525036	-0.903288631	-0.869751753	0.723447656	1.135201634	1.358406402	0.365255796
TG(20:0_20:0_18:3)	-0.907089324	-0.832035507	-0.647307262	-0.885504606	0.393295175	1.877540253	0.725095068	0.276006203
PC(34:2)	-0.828497097	-0.831013223	-1.009529173	-0.800552815	1.280427789	1.425033947	0.361930919	0.402199652
DGDG(18:2_18:2)	-0.827371868	-0.839690766	-1.089113641	-0.775216337	1.424760572	0.249272331	0.872669177	0.984690532
DG(18:1_18:2)	-1.06765915	-0.55733811	-0.763494032	-0.518598878	1.952338188	0.93731402	0.06903575	-0.051597788
TG(O−15:2_6:0_15:1)	-1.115691825	-0.573820906	-0.798908724	-0.554947987	1.901688705	0.908201384	0.041796648	0.191682705
TG(20:0_18:2_18:3)	-0.823687161	-0.842819224	-0.698114899	-0.905560978	0.106936659	1.267586285	1.614469576	0.281189742
TG(16:0_22:0_18:2)	-1.626732154	0.03444881	-0.641391453	-0.696388709	0.176889016	1.160043162	1.433037178	0.160094149
MG(O−15:1)	1.32090621	1.205541391	0.107426759	0.830994513	-0.643816416	-1.013217091	-1.014691203	-0.793144162
DG(O−15:1_24:0)	-0.2346119	-0.027676888	1.092791027	1.922499407	-0.369834594	-0.76851467	-0.914684164	-0.699968217
DGDG(18:0_18:2)	0.925377258	1.189294954	-0.110793282	1.196404442	-0.802893043	-0.237396906	-0.725161976	-1.434831447
SiE(18:2)	0.421661347	0.578305879	0.696632038	1.645203716	-0.303028702	-1.24593008	-1.045518103	-0.747326096
DG(O−27:6_20:3)	-0.916947467	-0.917089474	-0.915464498	-0.913045085	0.662616016	0.908991812	1.357790696	0.733148
TG(15:2_24:2_21:4)	-0.8768606	-1.025686496	-0.774948664	-1.003709843	0.737499632	1.122240667	1.144084392	0.677380914
PC(19:2_17:0)	-0.889814205	-0.7771385	-0.904352356	-0.836948362	1.531353437	0.126382835	0.564791529	1.185725621
PE(16:0_18:2)	-0.928166289	-0.922585172	-0.881030075	-0.909356119	1.301158829	1.061534053	0.485726407	0.792718367
WE(O−28:6_18:3)	-0.899389753	-0.945924206	-0.954199183	-0.825057431	1.405862033	0.857846421	0.497690482	0.863171636
TG(18:2_16:0)	-0.719186472	-0.715824205	-0.638788904	-0.746639436	-0.343298602	1.221381361	1.839752979	0.102603279
PC(16:2_18:3)	-0.714012487	-0.723814428	-0.687769296	-0.70664695	-0.665276157	1.644716136	0.671667442	1.18113574
TG(16:1_18:2_18:3)	-0.844674928	-0.814066295	-0.948739678	-0.844914932	0.081440616	1.491526556	1.018524326	0.860904333
TG(15:0_22:2_21:4)	-0.805210231	-0.797979247	-0.729648386	-0.78795989	-0.309051066	1.676507469	1.119883801	0.63345755
DG(18:0_18:2)	-0.931117218	-0.715954187	-0.775529409	-0.533191678	-0.038059935	1.548827789	1.525732997	-0.080708359
TG(18:0_20:0_18:2)	-1.175938302	-0.920420126	-0.304118111	-1.017660129	0.432853227	1.119668013	1.412702026	0.452913401

TG(16:0_18:0_18:1)	-0.947342701	-0.831347356	-0.200871518	-0.87189397	-0.383452057	0.580176878	1.876645173	0.77808555
Hex1Cer(d18:2_16:1)	-0.99939808	-0.882955741	-0.853854174	-0.969446441	1.191317702	0.7243256	0.836937012	0.953074123
PC(34:3)	-0.877394409	-0.877941904	-0.875389861	-0.876381803	1.557505569	0.331159207	1.009065759	0.609377442
DG(18:1_18:3)	-0.92381042	-0.865947741	-0.809168089	-0.919912594	1.215606202	0.120599808	0.987559863	1.195072971
TG(O−15:1_3:0_18:3)	-0.956767827	-0.897533909	-0.828542586	-0.935320146	1.233136132	0.347601758	0.99978735	1.037639228
TG(18:1_20:2_18:3)	-0.857022847	-0.858229386	-0.856979882	-0.858229386	0.530790669	0.192871772	1.158896139	1.54790292
TG(18:3_18:3_18:3)	-0.944171889	-0.966893193	-0.80779913	-0.953226779	0.689321534	1.331399175	0.801432014	0.849938267
TG(20:2_18:1_18:3)	-0.913456942	-0.948338135	-0.912532051	-0.948338135	0.934737744	0.838385974	0.805641494	1.143900051
TG(18:1_18:1_22:1)	-0.766665638	-0.691556343	-0.902142865	-0.9948426	0.715817418	1.192377093	1.481569509	-0.034556574
DG(O−26:6_20:3)	-0.910187587	-0.912244284	-0.912244284	-0.905414525	0.980407126	0.980912263	1.260622101	0.41814919
TG(15:0_22:1_21:4)	-0.79876069	-0.928055026	-0.924483823	-0.90473075	0.454422046	1.171294309	1.404475452	0.525838482
TG(16:0_18:2_18:3)	-0.380752904	-1.050143402	-1.245590408	-0.925625643	0.882858252	0.911068738	1.127358968	0.680826398
PE(17:0_16:0)	-1.121925396	-0.902874573	-0.7769274	-0.702135963	1.393038871	0.18637298	0.927290935	0.997160546
DG(O−17:1_20:0)	0.739943317	1.273494209	0.018610428	1.366100389	-0.64171026	-0.804465452	-0.970308923	-0.981663709
FA(16:0)	0.891378925	0.986699131	1.268941861	-0.227423949	0.004647981	-0.282163046	-1.321040451	-1.321040451
FA(18:0)	1.27642272	0.877768931	1.227683789	-0.196291344	-0.301838461	-0.604012662	-1.139866486	-1.139866486
WE(O−11:0_16:2)	0.885820045	1.487668747	0.71338716	0.432639522	-1.153130827	-0.610956113	-1.054241326	-0.701187209
PE(17:1_18:0)	0.288990768	0.680170524	1.745431751	0.695353879	-0.95375362	-0.850441534	-0.773830744	-0.831921023
PC(14:1_18:0)	0.430784269	0.668166247	1.728514118	0.621662837	-0.976536995	-0.845752675	-0.785757256	-0.841080545
DG(O−16:3_20:1)	0.451783369	0.58414077	1.72617729	0.691964278	-0.958957788	-0.814809725	-0.818322383	-0.861975811
TG(O−17:0_20:0_3:0)	1.205103802	1.038167589	0.418386499	0.984523715	-0.874294442	-0.918309982	-0.922288752	-0.931288429
d5−TG(36:6_20:2)	0.891789548	0.947655025	1.070108004	0.823345607	-0.933279907	-0.933141331	-0.933279856	-0.93319709
PMt(15:0_18:2)	0.61264416	1.240898532	0.89012259	0.935293229	-0.806715183	-0.850863408	-1.01068996	-1.01068996
PMt(17:0_18:2)	0.554636705	0.995717054	1.339399803	0.745650265	-0.79795331	-0.803218187	-1.017116165	-1.017116165
TG(16:1_18:1_18:2)	0.724610479	1.03355152	1.453686186	0.349591347	-0.899510353	-0.899170191	-0.883346513	-0.879412475
AcHexSiE (20:0)	0.625562112	0.353540641	1.918568921	0.353893725	-0.930964562	-0.832912099	-0.671431948	-0.81625679
TG(18:2_16:0_18:3)	0.091624402	0.40548158	2.012082541	0.594814742	-0.831746	-0.825627309	-0.67292672	-0.773703236
DG(P−16:1)	0.279465544	0.465847381	1.995846191	0.426043382	-0.713794835	-0.866243152	-0.894934097	-0.692230413
MG(17:3)	1.816752255	0.894032857	0.612514478	-0.356005404	-0.373444475	-0.852792423	-0.959541931	-0.781515357
d5−TG(38:5_18:3)	0.957558311	1.276533337	0.445089957	0.84867629	-0.365814895	-1.265489581	-0.921384766	-0.975168653
MG(O−25:6)	0.70152601	1.342133979	1.08414684	0.492540964	-0.875190766	-0.990705466	-0.845129626	-0.909321936

PMt(18:0_18:2)	0.323666264	0.512525894	1.827554093	0.484836278	-0.479490776	-0.340187057	-1.164452348	-1.164452348
TG(22:0_18:2_18:2)	0.400422742	1.032419392	1.400456387	0.705048221	-1.050687819	-0.758961042	-0.598608648	-1.130089233
TG(O−14:0_21:0_2:0)	0.661445103	0.869381411	1.076286236	1.050118405	-0.679179218	-0.700934944	-1.150816317	-1.126300676
PMt(18:1_18:3)	0.511994395	0.963519507	1.544764839	0.32072986	-0.273146748	-0.753724203	-1.157068824	-1.157068824
DGDG(8:0_22:1)	0.705728731	1.52214002	0.043284917	0.777368376	-0.779870427	-0.875068215	-1.482247597	0.088664195
DG(P−18:4_18:1)	0.465636855	1.108040364	0.344945722	0.782283802	0.676854868	-1.199912322	-0.562124152	-1.615725137
PA(19:2_18:2)	0.610006587	1.151768872	0.784347278	1.018674548	-0.60026472	-0.499219608	-1.232656479	-1.232656479
TG(18:0_18:1_18:3)	0.975184646	0.980831959	0.937627569	0.666153495	-0.484091184	-1.304208474	-1.246075951	-0.525422061
DG(O−16:2_16:0)	-0.926975308	-0.892769698	-0.73822414	-0.856363303	0.024493799	0.74357132	1.250737488	1.395529842
d5−TG(34:5_18:3)	-0.792843485	-0.794507302	-0.818822497	-0.759190679	-0.106308011	1.86968395	0.659089896	0.742898129
TG(16:0_16:0_18:3)	-0.833784685	-0.829068018	-0.805872342	-0.833205126	-0.15859842	1.450254071	1.25493333	0.75534119
PE(17:2_16:0)	-0.880171472	-0.830330435	-0.735815626	-0.831548395	-0.168023837	1.597271277	0.838432936	1.010185552
TG(P−19:4_24:8_3:0)	-0.827271129	-0.884083237	-0.871614884	-0.925150989	1.534531351	0.633096437	1.033801503	0.306690947
TG(P−19:4_24:7_3:0)	-0.746950307	-0.752947453	-0.760007047	-0.734532036	1.431216138	-0.514452481	1.417375511	0.660297675
DG(18:2_18:3)	-0.900249369	-0.882467859	-0.84504949	-0.89627381	0.803348968	1.183704418	1.345423253	0.191563889
MG(P−18:4)	-0.898193954	-0.915028237	-0.872464624	-0.901445389	0.4325514	1.297293732	1.234701753	0.622585319
DG(16:0_18:2)	-0.98487679	-0.715712993	-1.000354834	-0.728252224	0.944461439	1.12352225	1.353641214	0.007571938
PE(17:2_18:2)	-1.022580481	-0.943927154	-0.780314301	-0.929890904	1.025236226	0.544670115	0.946980689	1.15982581
